# STAT3/SOCS3 axis contributes to the outcome of salmonid whirling disease

**DOI:** 10.1371/journal.pone.0234479

**Published:** 2020-06-15

**Authors:** Mona Saleh, Adina Friedl, Mitaly Srivastava, Hatem Soliman, Christopher J. Secombes, Mansour El-Matbouli

**Affiliations:** 1 Clinical Division of Fish Medicine, Department for Farm Animals and Veterinary Public Health, University of Veterinary Medicine Vienna, Vienna, Austria; 2 Scottish Fish Immunology Research Centre, School of Biological Sciences, University of Aberdeen, Aberdeen, Scotland, United Kingdom; INRA, FRANCE

## Abstract

There are differences in disease susceptibility to whirling disease (WD) among strains of rainbow trout. The North American strain Trout Lodge (TL) is highly susceptible, whereas the German Hofer (HO) strain is more resistant. The suppressor of cytokine signaling (SOCS) proteins are key in inhibiting cytokine signaling. Their role in modulating the immune response against whirling disease is not completely clear. This study aimed at investigating the transcriptional response of SOCS1 and SOCS3 genes to *Myxobolus cerebralis* along with that of several upstream regulators and immune response genes. *M*. *cerebralis* induced the expression of SOCS1, the IL-6-dependent SOCS3, the anti-inflammatory cytokine IL-10 and the Treg associated transcription factor FOXP3 in TL fish at multiple time points, which likely caused a restricted STAT1 and STAT3 activity affecting the Th17/Treg17 balance. The expression of SOCS1 and the IL-6-dependent SOCS3 was induced constraining the activation of STAT1 and STAT3 in TL fish, thereby causing Th17/Treg17 imbalance and leaving the fish unable to establish a protective immune response against *M*. *cerebralis* or control inflammatory reactions increasing susceptibility to WD. Conversely, in HO fish, the expression of SOCS1 and SOCS3 was restrained, whereas the expression of STAT1 and IL-23-mediated STAT3 was induced potentially enabling more controlled immune responses, accelerating parasite clearance and elevating resistance. The induced expression of STAT1 and IL-23-mediated STAT3 likely maintained a successful Th17/Treg17 balance and enabled fish to promote effective immune responses favouring resistance against WD. The results provide insights into the role of SOCS1 and SOCS3 in regulating the activation and magnitude of host immunity in rainbow trout, which may help us understand the mechanisms that underlie the variation in resistance to WD.

## Introduction

*Myxobolus cerebralis* infects several salmonid species causing whirling disease (WD). Salmonids show variable susceptibility to WD and rainbow trout is the most susceptible species. The severity of the infection differs broadly between species [1–3] and between strains [4–10]. The North American strain Trout Lodge (TL) is highly susceptible to WD, whereas the German Hofer (HO) strain is more resistant [4–5]. The reasons for the differences in susceptibility are not completely understood and the mechanisms of the varying levels of resistance to WD need further exploration. *M*. *cerebralis* is a myxozoan parasite that alternates between salmonid fish and the oligochaete host *Tubifex tubifex*. After the intake of *M*. *cerebralis* spores by *T*. *tubifex*, they develop in the intestine and release triactinomyxon spores, which infect salmonid fish [11–12]. The development of *M*. *cerebralis* in the epidermis of rainbow trout appears hampered as some of the parasites are eliminated, probably by cellular and humoral responses in the fish's skin [11–13]. Conversely, the parasites are privileged from host immune reactions during migration through peripheral nerves and the central nervous system [12]. Several transcriptional studies have provided important insights into the mechanisms utilized by myxozoan parasites to evade the fish immune system [14]. Some of these studies aimed to reveal the mechanisms involved in disease resistance [6–10]. Dionne et al. [15] reported that different major histocompatibility complex (MHC) alleles influence the levels of susceptibility and resistance of salmonids. Quantitative genetic and genome wide mapping studies have emphasized that a single quantitative trait locus (QTL), Omy9, may explain the phenotypic variance of WD resistance in rainbow trout [16–17]. However, this does not rule out that other genes may exhibit critical functions in the fish response to *M*. *cerebralis* infection that are not present in the specific Omy9 region [10].

The regulation of the expression of metallothionein and other genes associated with immunity and inflammation is likely under the control of STAT3 [18]. Indeed, STAT3 was the only gene with constantly elevated expression values in the resistant strain but was unaffected in the susceptible fish and was suggested a good candidate for future studies of resistance mechanisms against *M*. *cerebralis* [10]. Elevated STAT3 expression in the German strain may promote resistance by generating a specific class of T helper cells, Th17, since STAT3 is a critical component in the differentiation of Th17 from naive CD4^+^ T cells [10, 19]. Th17 cells have defensive roles retaining and guarding the mucosal surface against microbes by producing IL-17 and other cytokines, which can help reduce pathogen burden at epithelial barriers and mucosal sites [20–25]. IL-17 has a protective function to primary infections against extracellular pathogens, intracellular invaders and fungal infections [20, 12, 25, 26]. However, the Th17 response can also be a double edged sword and the equilibrium linking protection and pathology may influence the outcome of the infection [25]. Th17 cells can amend their differentiation to generate either pro-inflammatory or regulatory (Treg17) cells. Additionally, some pathogens manipulate regulatory T cells to immunosuppress the host and so potentiate their own survival [27]. Functional studies performed thus far show a high level of conservation of fish and mammalian T cell responses are [28].

Using recently established monoclonal antibodies, CD4^+^ T-cell populations were identified in rainbow trout [29]. Effective local and systemic immune reactions and proper activation of T lymphocytes were suggested to drive the immune response mediating resistance against *M*. *cerebralis* in HO fish [13]. On the other hand, alteration of the leukocyte populations with early augmented local cellular responses in TL fish likely promotes excessive local inflammatory reactions and leads to subsequent host tissue damage, supporting parasite invasion and development [13]. Despite increased immune responses, the susceptible strain is unable to exhibit protective immune responses after infection with *M*. *cerebralis* [9–10, 13]. Investigating the occurrence of Th17 (pro-inflammatory) and Treg17 (regulatory) cells in susceptible and resistant fish strains to WD may enhance our understanding of the impact of Th17/Treg17 balance on host responses and resistance mechanisms in rainbow trout [13]. While the pro-inflammatory Th17 are activated in mammals by interleukin 23 (IL-23) and IL-1β, Treg17 cells are induced by IL-6 and transforming growth factor-beta (TGF-β) [24, 30, 31]. Treg17 produce IL-17 and IL-10 offering a novel regulatory T cell population for modulating immune responses [32]. The modulation of genes encoding immunosuppressive molecules including suppressor of cytokine signaling (SOCS) proteins SOCS1 and SOCS3 may suggest immune evasion strategies of myxozoan parasites [33–35]. They are key in inhibiting cytokine signaling via the JAK/STAT pathway. However, their role in modulating the immune response against whirling disease is not completely clear.

In the current study, a well-established model of WD infection was used to explore whether the STAT3/SOCS3 axis modulates Th17/Treg17 responses, thereby influencing disease outcome in rainbow trout. All genes investigated in the present study are part of the innate immune system, however some also have dual roles in the adaptive immune system. Expression patterns of relevant immune-related genes was investigated after *M*. *cerebralis* exposure in the two rainbow trout strains, the susceptible TL and the more resistant HO, to explore if these are modulated/involved in the host immune response observed during WD. The obtained results may help us to understand mechanisms underlying the varying levels of resistance to *M*. *cerebralis* in both trout strains.

## Materials and methods

### Fish and infective triactinomyxon spores of *M*. *cerebralis*

Specific-pathogen-free fish (SPF), the German Hofer strain (HO), with a degree of resistance to WD and the highly susceptible North American Trout Lodge stain (TL) were reared in separate tanks at 14±2°C with dechlorinated aerated spring water in our wet laboratory[13]. They were fed a commercial trout diet ad libitum twice daily throughout the trial Laboratory cultures of *Tubifex tubifex* oligochaetes were maintained at 14°C and exposed to *M*. *cerebralis* spores that were isolated and purified from laboratory-infected rainbow trout. The waterborne triactinomyxon stages (TAMs) were collected using a polyamide filter (15 μm). The number of TAMs was assessed for the experiment by filtering the TAMs two times per week [13].

### Experimental infection of rainbow and collection of samples

The experimental infection and sample collection has been described and published by Saleh et al. [13]. In brief, SPF fish (3–4 cm, 90 d old) of the TL and HO strains were kept in replicate tanks (60 fish/group). An equal number of fish was used as a negative unexposed control group. For experimental infection, replicate groups were exposed to freshly filtered TAM spores (1,000 spores/fish) for 1h with the water flow off. Fish were then transferred into separate aquaria at 15ºC and fed daily with a commercial trout diet and observed by well-trained animal care personnel. The fish were observed three times a day for immediate removal and euthanizing of moribund fish when required (observation of uncoordinated swimming and/or lethargy).

This study was carried out in strict accordance with the recommendations in the Guide for the Care and Use of Laboratory Animals of the National Institutes of Health. The protocol was approved by the Committee on the Ethics of Animal Experiments of Vienna University of Veterinary medicine (BMWFW-68.205/0167-WF/V/3b/2017). All experiments were performed in accordance with relevant guidelines and regulations to minimize suffering of the fish.

Humane endpoint euthanasia was applied by immersion in 0.05% (w/v) MS-222 (Sigma Aldrich, Vienna, Austria) at determined time points. No fish died during the experiment or prior to the determined endpoint euthanasia at the specified time points. The caudal fin was collected from five individuals of each of the four experimental groups (i.e., exposed and nonexposed fish from both HO and TL strains) at the following early time points after *M*. *cerebralis* exposure: 2 h, 4 h, 8 h, 12 h, 24 h, 2 d, 4 d. The samples were kept separately in RNA*later* at 4°C overnight then stored at -20°C until used for RNA isolation.

### DNA extraction and pathogen load

The severity of *M*. *cerebralis* infection at all time points was previously described and published [13]. Briefly, DNA was extracted from CF tissues, utilizing the DNeasy blood and tissue kit following the manufacturer's instructions (Qiagen, Hilden, Germany). A TaqMan quantitative polymerase chain reaction (qPCR) assay was performed according to Kelley et al. (2004) to quantify the 18S rDNA gene copy number of *M*. *cerebralis* in each sample at different time points. The insulin growth factor-I gene was amplified as a host reference. Significant difference between the HO and TL data were estimated at each time point with the two-tailed *t* test.

### RNA isolation and complementary DNA synthesis

Total RNA was extracted from the caudal fin tissues using RNeasy® Mini Kits (QIAGEN, Hilden, Germany) in accordance with the manufacturer’s instructions. An on-column DNase digestion step was performed in order to eliminate any contamination with residual DNA. Total RNA concentrations were measured using a Nanodrop 2000c spectrophotometer (Thermo Fisher Scientific, Wilmington, USA). RNA quality was assessed by agarose gel electrophoresis to confirm its purity and absence of gDNA. Isolated RNA samples were reverse transcribed into complementary DNA (cDNA) using an iScript™ cDNA Synthesis Kit (Bio-Rad, Munich, Germany) in accordance with the manufacturer’s instructions. The synthesized cDNA was diluted with nuclease-free water, aliquoted and stored at −20°C until required.

### Quantitative real-time PCR

Real-time PCR was carried out to assess the expression levels of STAT3, SOCS3 and other genes could be involved in shaping host Th17 responses in infected and control fish. The GenBank accession numbers for all genes and gene paralogs (selected based on previous observations following myxozoan infections) and specific primers used in the assay are presented in Table 1. For each PCR primer pair, primer efficiencies were assessed using a serially 10 fold diluted DNA template. The β-actin gene was used as reference gene. This gene was reported suitable as a housekeeping gene during WD infection and used in numerous previous gene expression studies (6–10). The relative fold change of the genes under study was determined using the CFX96 Touch Real-Time PCR detection system (Bio-Rad, Munich, Germany). The real-time PCR reactions were performed in duplicate 10μl reaction volumes. Each PCR reaction contained 1μl of 1:10-fold diluted cDNA, 1× SsoAdvanced Universal SYBR Green Supermix (Bio-Rad, Munich, Germany), 0.1 μM of each primer, and DEPC-treated sterile distilled water (Bio-Rad, Munich, Germany). In addition to a no template control, a negative no reverse transcriptase control was included to assess for the presence of genomic DNA contamination.

**Table 1:** Rainbow trout oligonucleotides used in this study.

Gene Name	Gene ID (NCBI)	Forward primer (5’ to 3’)	Reverse primer (5’ to 3’)	Size	Ref.	BaselineCT
IL-10A	100136835	GGATTCTACACCACTTGAAGAGCCC	GTCGTTGTTGTTCTGTGTTCTGTTGT	119	[34]	32.35
IL-6	100136689	CCTTGCGGAACCAACAGTTTG	CCTCAGCAACCTTCATCTGGTC	288	[34]	34.71
IL-23p19a	110492907	ACCTAAGAGCAGATTCAATGCCTTG	TCTTCCCAGCTCTTCACTTCCTG	210	[60]	34.38
IL-17A/F2a	100136642	CGTGTCGAAGTACCTGGTTGTGT	GGTTCTCCACTGTAGTGCTTTTCCA	212	[34]	29,62
IL-17C1	100462681	CTGGCGGTACAGCATCGATA	GAGTTATATCCATAATCTTCGTATTCGGC	138	[34]	31.78
FOXP3-1	100653439	CCCAGAACCGAGGTGGAGTGT	TGACGGACAGCGTTCTTCCA	319	[34]	28.13
SOCS1a	100272210	GATTAATACCGCTGGGATTCTGTG	CTCTCCCATCGCTACACAGTTCC	136	[34]	27.07
SOCS3a	100272212	CACAGAGAAACCGTTAAAAGGACTATCC	AAGGGGCTGCTGCTCATGAC	228	[34]	24.18
STAT1	100136755	GACCAGCGAACCCAAGAACCTGAA	CACAAAGCCCAGGATGCAACCAT	319	[61]	22.82
STAT3	100136756	GAATGAAGGGTATATTCTGG	TCCCACTGATGTCCTTTTCC	152	[10]	23.96
RORγ	100528059	ACAGACCTTCAAAGCTCTTGGTTGTG	GGGAAGCTTGGACACCATCTTTG	262	[34]	25.90
IL-21	115205151	CAACAGTGTGATGTCGAACGCTC	CCTTGGCAGACTGTTTTCTCTC	207	[34]	31.69
B-actin	100135845	CAGGCATCAGGGAGTGATG	GTCCCAGTTGGTGACGATG	127	[10]	15.37

The PCR cycling conditions consisted of a 5 min cDNA denaturation at 95°C, followed by 45 cycles of 15 s denaturation at 95°C, 15 s annealing at 55°C and 15 s elongation at 72°C. A melting-point curve analysis was implemented starting from 55°C with an increase of 0.5°C every 10 s up to 95°C to check for non-specific binding. The normalized expression of the samples was assessed using CFX Maestro Software (Bio-Rad Laboratories Inc., Munich, Germany). For each gene, the relative fold change was calculated using the comparative CT method (2^-∆∆C^ T). To identify differentially expressed genes, a nonparametric Mann-Whitney U-test was used to compare between *M*. *cerebralis* exposed and unexposed control fish for each strain. Further, gene expression significantly differing between exposed and unexposed rainbow trout in one strain was tested for significant differences between strains using a Mann-Whitney U-test. For both within and between strain comparisons, *p* values less than 0.05 were considered significant.

## Results

### *Myxobolus cerebralis* infection prevalence and parasite burden

**Fig 1:**
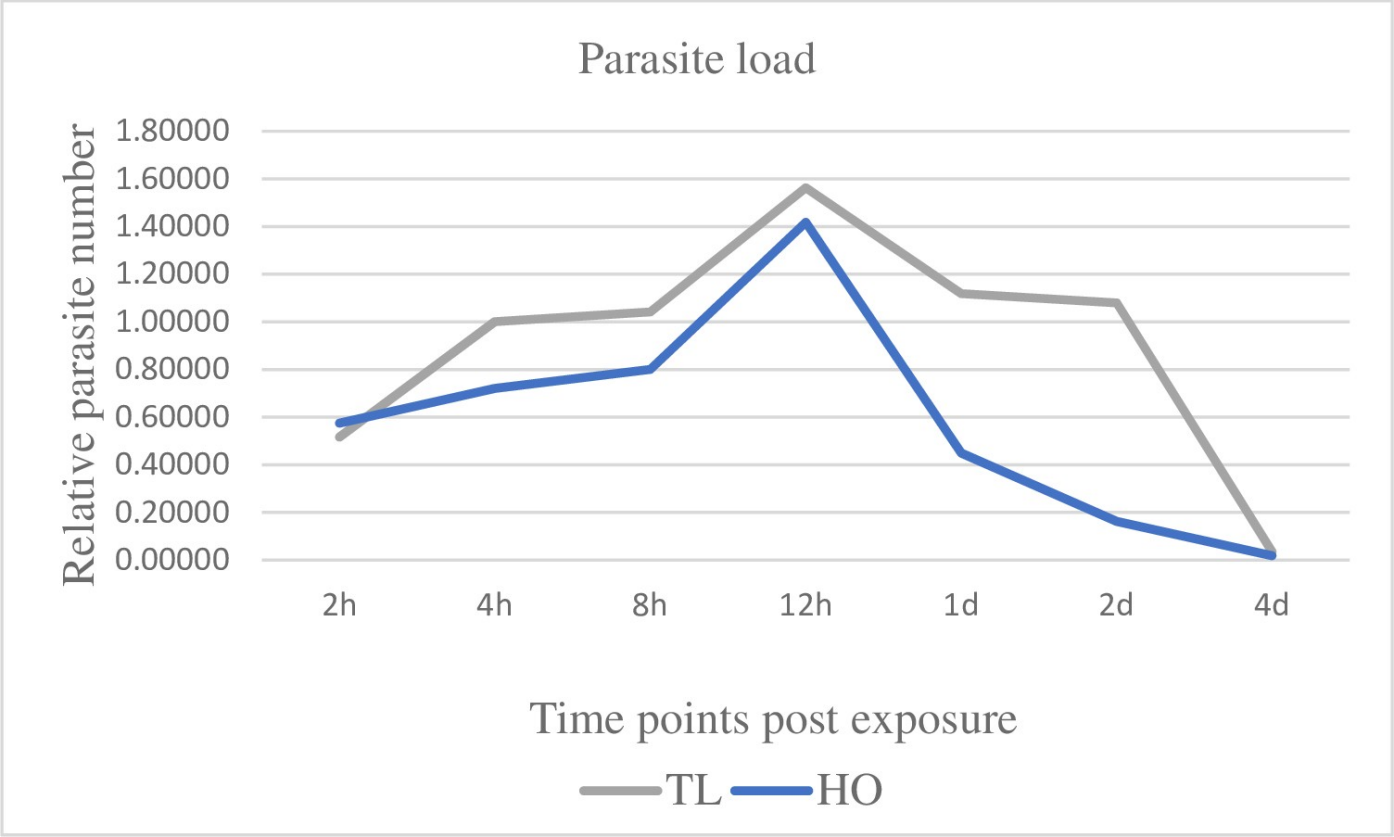
Pathogen load in caudal fin tissues of susceptible Trout Lodge (TL) and resistant Hofer (HO) rainbow trout post exposure to *Myxobolus cerebralis*. Two days post-exposure (2dpe), the HO strain exhibited a markedly declined parasite count compared to the TL strain. The vast majority of *M*. *cerebralis* have migrated from the fins at 4 dpe, and are not quantifiable in skin tissue thereafter.

The infection prevalence was 100% (10/10 fish sampled) after exposure of TL and HO fish to the parasite. When compared with the HO strain, the parasite number was higher in the TL strain at all time points (Fig 1). The parasite load in TL at 2 hpe, was not significantly different relative to HO fish. By 12 hpe, both TL and HO fish displayed the highest numbers of parasite load. At 1 dpe, parasite intensity in HO fish significantly declined > 3-fold (*p* = < 0.05), compared to its maximum value at 12 hpe. The HO strain displayed a major decrease of parasite number at 2 dpe (> 7-fold) compared to its 12 hpe value, with TL parasite number now > 5-fold higher compared to HO. At all time points between 2 hpe and 2 dpe, the HO strain showed lower parasite values compared to the TL strain. At 4 dpe, *M*. *cerebralis* intensity significantly declined in both trout strains. The parasite was not detectable in control samples of either strain at all-time points.

### Host gene expression changes of signal transducer and activator of transcription genes STAT1 and STAT3 in response to *Myxobolus cerebralis*

**Fig 2:**
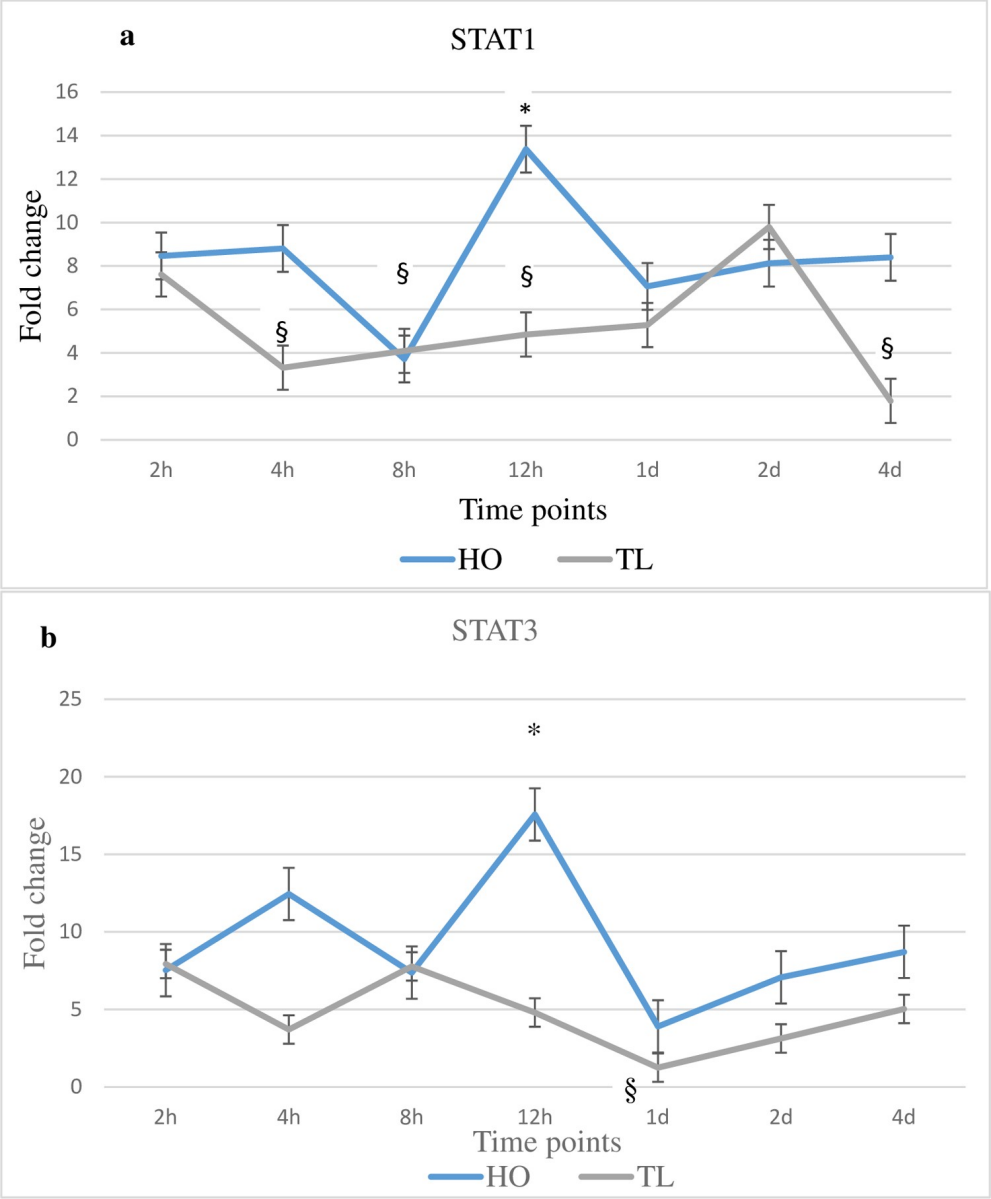
Transcription levels of STAT1 and STAT3. The figure shows the mean± SD (n = 5) fold change of the genes in *Myxobolus cerebralis* exposed fish; resistant Hofer (HO) and susceptible Trout Lodge (TL) fish. Lines are the mean fold change of STAT1 and STAT3. and ROR-γ measured at different time points post exposure hpe/dpe. All HO and TL means are presented normalized to HO and TL timed levels of control fish ± SD (n = 5). * = significantly higher and ^§^ = significantly lower to values in non-exposed fish (p < 0.05).

Expression changes for the investigated genes were studied up to 4 d post *M*. *cerebralis* exposure (dpe). The expression of STAT1 and STAT3 increased in HO at several time points, as compared to unexposed controls and TL (Fig 2). Conversely, in TL, the expression of STAT1 and STAT3 generally decreased at multiple time points (Fig 2). The expression of STAT1 was upregulated at five time points in HO, showing a significant increase (2.2-fold) at 12 hpe (*p* = < 0.05 compared to unexposed control and TL). For TL fish, STAT1 expression significantly decreased at 4, 8 and 12 hpe as well as at 4 dpe compared to unexposed controls. The expression of STAT3 was increased at several time points, with the expression of STAT3 at 12 hpe significantly higher compared to the control (2.2-fold) and TL (3.9-fold) fish. On the other hand, the expression of STAT3 was unchanged in TL at several early time points and was significantly decreased at 1 dpe compared to unexposed controls.

### Host gene expression changes of suppressor of cytokine signalling genes SOCS1 and SOCS3 in response to *Myxobolus cerebralis*

**Fig 3:**
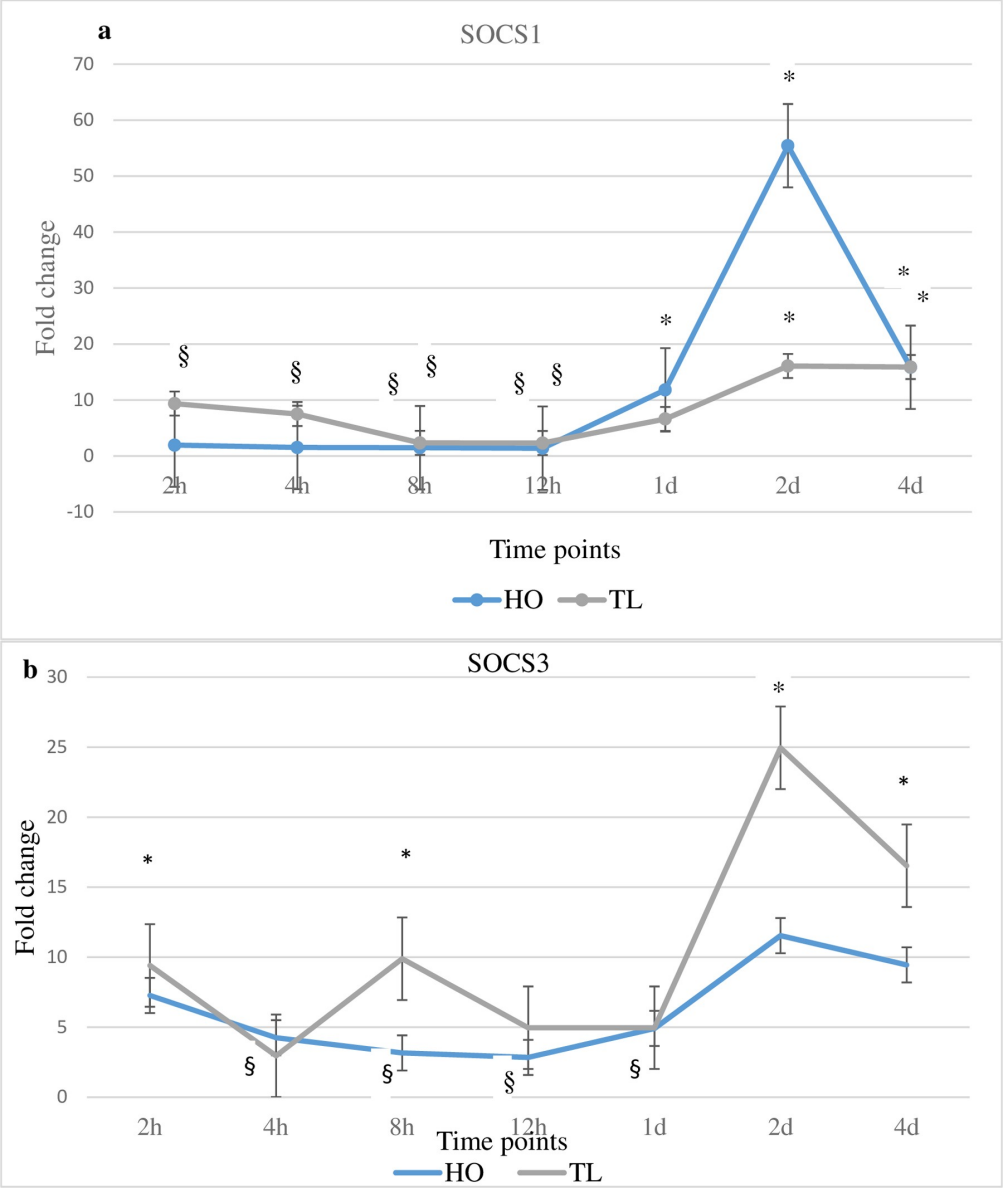
Transcription levels of SOCS1 and SOCS3. The figure shows the mean fold change of the genes in *M*. *cerebralis* exposed HO and TL fish. Lines are the mean fold change of IL-17A, IL-17C, IL-21 and ROR-γ measured at different time points post exposure hpe/dpe. All HO and TL means are presented normalized to HO and TL timed levels of control fish ± SD (n = 5). * = significantly higher and ^§^ = significantly lower to values in non-exposed fish (p < 0.05).

The expression of the SOCS genes SOCS1 and SOCS3 increased in TL at the majority of time points when compared with unexposed control or HO fish (Fig 3). On the other hand, the expression of SOCS1 and SOCS3 was consistently downregulated in HO fish at the early time points. Specifically, the expression of SOCS1 increased for TL at multiple time points and showed significant increases at 2 and 4 dpe compared to unexposed control fish. For HO, the expression of SOCS1 was significantly decreased at 2, 4, 8 and 12 hpe but showed significant increases at 1, 2 and 4 dpe. The most significant increase (9.7-fold) occurred at 2 dpe compared to unexposed control. However, at that time point SOCS1 expression in TL fish was 1.4-fold higher compared to HO fish. Similarly, SOCS3 was significantly increased in TL fish at 2 and 8 hpe as well as at 2 and 4 dpe compared to unexposed control fish, showing significant upregulation at 8 hpe as well as at 2 and 4 dpe compared to HO fish. In contrast, the expression of SOCS3 decreased significantly in HO at 4, 8 and 12 hpe, as well as at 1 dpe, compared to unexposed control fish. At 2 dpe, as for SOCS1, SOCS3 expression in TL fish was significantly higher compared to HO and unexposed control fish.

### Host gene expression changes of Th-17 signature genes IL-17A, IL-17C, IL-21 and ROR-γ in response to *Myxobolus cerebralis*

**Fig 4:**
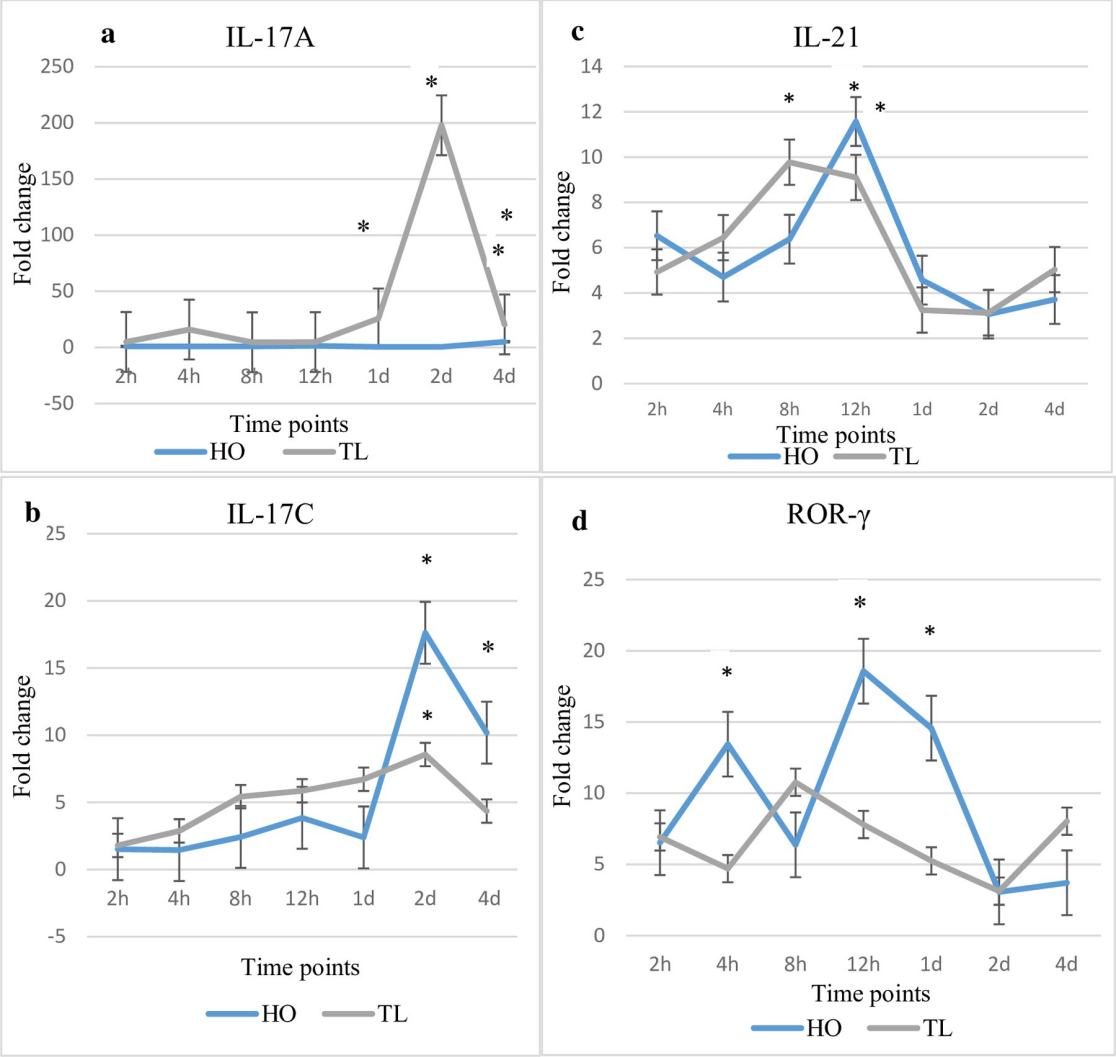
Transcription levels of IL-17A, IL-17C, IL-21 and ROR-γ. The figure shows the mean fold change of the genes in *M*. *cerebralis* exposed HO and TL fish. Lines are the mean fold change of IL-17A, IL-17C, IL-21 and ROR-γ measured at different time points post exposure hpe/dpe. All HO and TL means are presented normalized to HO and TL timed levels of control fish ± SD (n = 5). * = significantly higher and ^§^ = significantly lower to values in non-exposed fish (p < 0.05).

While the expression of IL-17A prominently increased in TL, the expression of IL-17C increased in one or both strains at several time points (Fig 4). In TL, the expression of IL-17A increased significantly at 1, 2 and 4 dpe compared to unexposed control fish, with a prominent upregulation (20.5-fold) at 2 dpe. In contrast, for HO fish, IL-17A expression remained unchanged at all time points except for a significant upregulation (3.1-fold) at 4 dpe. IL-17C was increased in TL fish at several time points showing a significant increase at 2 dpe compared to unexposed control and HO fish. For HO, IL-17C increased significantly at 2 and 4 dpe observing the highest upregulation at 2 dpe with a 5.4-fold increase compared to control fish.

The expression of IL-21 increased significantly at 8 hpe in TL fish and at 12 hpe in both TL fish and HO fish compared to unexposed control fish. On the other hand, while ROR-γ expression showed insignificant changes in TL fish compared to unexposed control, its expression increased at 12h (2.1-fold) and 1d (5.9-fold) in HO fish compared to TL fish and unexposed control fish.

### Host gene expression changes of Treg cell signature molecules FOXP3 and IL-10 in response to *Myxobolus cerebralis*

**Fig 5:**
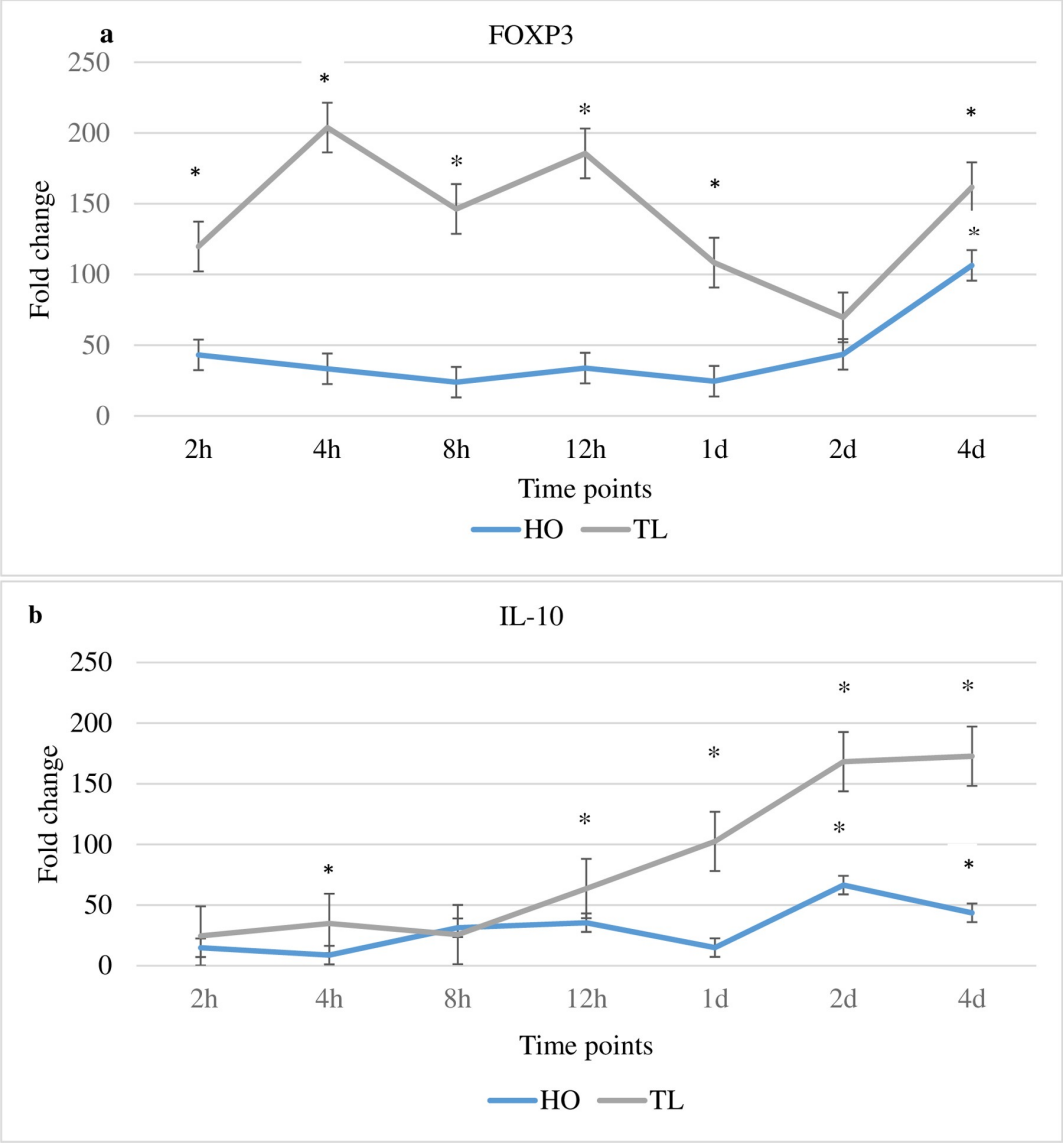
Transcription levels of FOXP3 and IL-10. The figure shows the mean fold change of the genes in *M*. *cerebralis* exposed HO and TL fish. Lines are the mean fold change of FOXP3 and IL-10 measured at different time points post exposure hpe/dpe. All HO and TL means are presented normalized to HO and TL timed levels of control fish ± SD (n = 5). ± SD (n = 5), * = significantly higher and ^§^ = significantly lower to values in non-exposed fish (p < 0.05).

The expression of FOXP3 and IL-10 was increased in TL fish, at the majority of time points compared to unexposed controls and HO fish (Fig 5). Conversely, for HO fish, the expression of FOXP3 and IL-10 was mostly unchanged or decreased at multiple time points. Specifically, the expression of FOXP3 for TL was increased significantly at 2, 4, 8 and 12 hpe as well as at 1 and 4 dpe compared to unexposed control and HO fish. Compared to HO fish, FOXP3 expression in TL fish was increased at the majority of time points. The expression of FOXP3 was significantly increased for HO at 4 dpe, but was unchanged or decreased compared to control fish at all other times. There were increases of the expression of IL-10 for TL fish at multiple time points compared to unexposed control and HO fish. Significant increases were observed at 4 and 12 hpe and at 1, 2 and 4 dpe compared to unexposed control and HO fish. When compared with transcript levels in HO fish, the expression of IL-10 was higher for TL at several time points. On the other hand, for HO, the expression of IL-10 exhibited significant increases at 2 and 4 dpe compared to unexposed control fish, however, at both time point, the TL transcript level was higher compared to HO fish.

### Host gene expression changes of Th-17 regulatory cytokines IL-6 and IL-23p19a in response to *Myxobolus cerebralis*

**Fig 6:**
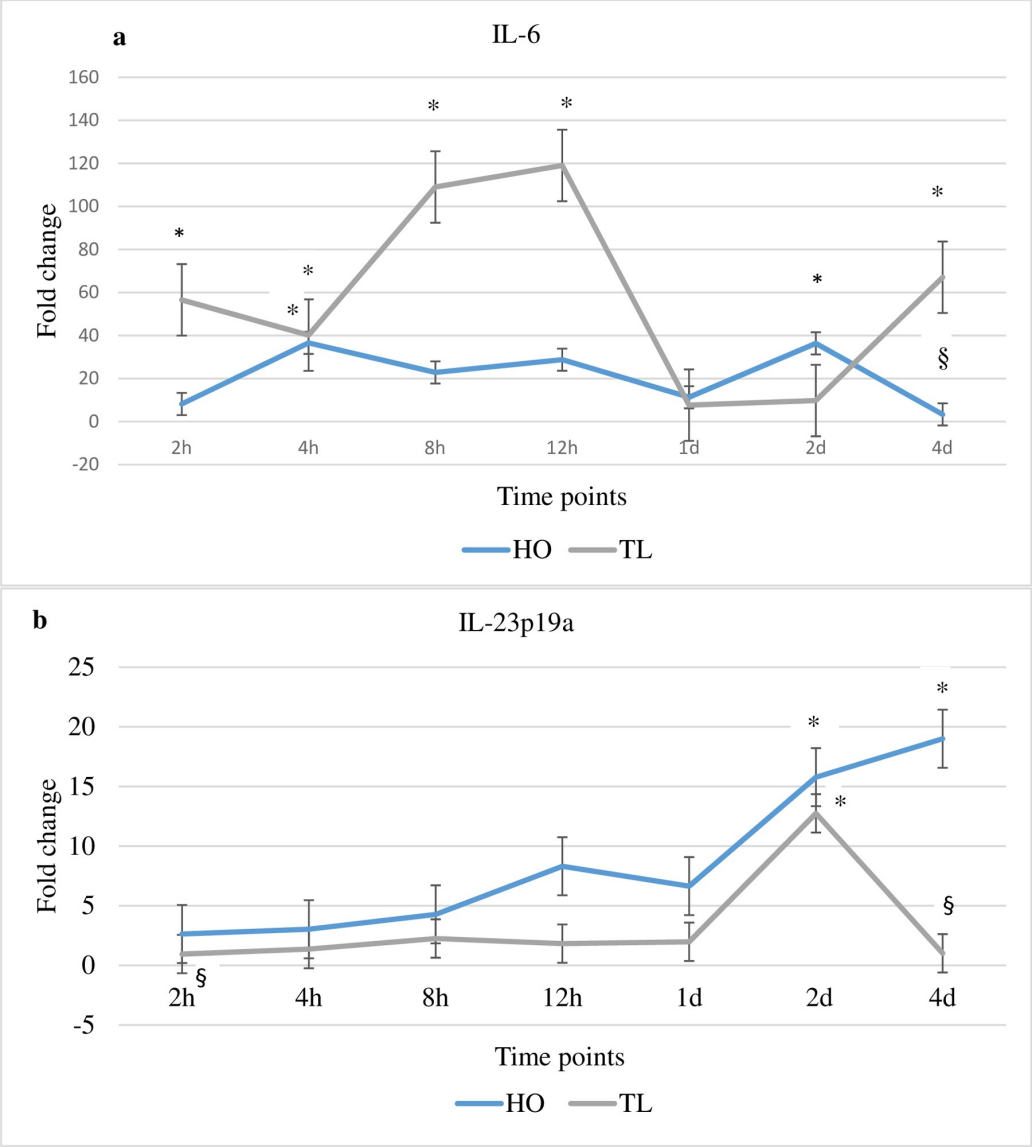
Transcription levels of IL-6 and IL-23p19. The figure shows the mean fold change of the genes in *M*. *cerebralis* exposed HO and TL fish. Lines are the mean fold change of IL-6 and IL-23p19 measured at different time points post exposure hpe/dpe (hours/days post exposure). All HO and TL means are presented normalized to HO and TL timed levels of control fish ± SD (n = 5). * = significantly higher and ^§^ = significantly lower to values in non-exposed fish (p < 0.05).

Analysis of the expression of the Th-17 regulatory cytokines IL-6 and IL-23p19a during WD suggests that these molecules may exert different roles in tuning Th-17 responses in both fish strains (TL and HO) studied (Fig 6). The expression of IL-6 in TL fish was increased at several time points compared to unexposed controls and HO fish. For HO, the expression of IL-23p19a was increased at various time points compared to unexposed controls and TL fish. Specifically, the expression of IL-6 in TL fish was significantly upregulated at 2, 4, 8 and 12 hpe as well as at 4 dpe when compared with unexposed control fish and/or HO fish. In contrast, in HO fish IL-6 expression was decreased at several time points, significantly so at 4 dpe. However, at 4 hpe and 2 dpe it was significantly increased compared to unexposed controls. The expression of IL-23p19a was significantly increased for HO at 2 and 4 dpe compared to unexposed control fish. When compared with TL fish, the expression of IL-23p19a showed increased values at the majority of time points. Significant IL-23p19a transcript levels were observed in HO at 4 hpe and 4 dpe compared to TL and unexposed control fish. On the other hand, for TL fish, the expression of IL-23p19a was a significantly increased at 2 dpe, although it was unchanged or decreased at the majority of time points compared to HO and unexposed control fish.

## Discussion

Since gene modified fish and functional tools are limited, gene expression studies of fish immune responses can give insights into the mechanisms and signalling pathways underlying host responses and invasion strategies of myxozoans, and can reveal relevant host-parasite interactions [34]. In this study we examined if *M*. *cerebralis* modulates the SOCS3/STAT3 axis thereby shaping host Th17 responses and determining WD disease outcome in rainbow trout, by determining the transcript levels of relevant immune genes and cytokines in two lines of rainbow trout, the susceptible TL and the more resistant HO.

The Th17 cells signature genes IL-17A, IL17c, IL-21 and ROR-γ were differentially modulated for one or both strains after *M*. *cerebralis* exposure indicating the involvement of these cells in the immune defense against this parasite. While the expression of IL-17A prominently increased in TL, the expression of IL-17C was also increased in HO. Th17 cells are protective cells, which maintain and guard the mucosal surface against microbial populations by producing IL-17 and other cytokines to help reduce pathogen loads at epithelial barriers [20–25]. Furthermore, IL-17 demonstrates a protective function for in immunity to primary infections against extracellular pathogens, intracellular invaders and fungal infections [26]. Although FOXP3^+^ Treg cells suppress other immune effector cells, they can produce IL-17A in inflammatory conditions [36]. Thus, IL-17A producing effector/Treg cells can contribute to excessive inflammation causing host tissue damage during infection [36]. In fact, in TL, the greatest upregulation of IL-17A expression occurred at 2 dpe correlating with the highest parasite load compared to HO as previously reported [13]. This may suggest the stimulation and recruitment of IL-17A producing effector/Treg cells on mucosal sites. However, further investigations are required to confirm this issue.

The modulation of FOXP3 in both strains was consequently investigated. The increasing expression of FOXP3 in TL suggests the induction of IL-17A producing Treg cells (IL-17A^+^ FOXP3^+^ Treg), which may likely contribute to excessive inflammatory responses impairing host immunity and supporting *M*. *cerebralis* development. Pathogen mediated chronic immunopathologies have been linked to dysregulated Th responses involving a complex interplay between stimulatory and suppressive immune signals leading to pathology rather than parasite clearance [37, 38]. FOXP3 was upregulated in rainbow trout infected with the myxozoan parasite *Tetracapsuloides bryosalmonae*, the causative agent of proliferative kidney disease (PKD). Together with increased expression values of IL-6 and TGF-β, the authors suggested that the lymphocytic character of PKD indicates a Th17-like activity [34]. Given the functional capabilities of FOXP3 and IL-17C, they were suggested to establish a counterbalance in an attempt to sustain tolerance [39]. Th17 cells may exhibit plasticity, expressing cytokines typical of other lineages in response to infections, ending in a transdifferentiation of Th17 cells into Tregs or vice versa [27, 40]. Treg cells activity increases in various parasitic infections including malaria and leishmaniasis [27]. Treg cells help control the immune response, but in some cases this excessive regulatory control allows parasite replication without restraint and compromises the host [27]. Functional studies performed thus far show a high level of conservation of these phenotypes [28]. Thus, in this study, the expression of IL-17A and FOXP3 was markedly increased in TL suggesting the possibility of transdifferentiating of FOXP3^+^ Treg cells into IL-17A producing FOXP3 cells. This probably stimulates inflammatory responses, host tissue damage enhancing the development and manifestation of *M*. *cerebralis* and suggests an inability to establish a protective Th17 response. On the other hand, the expression of IL-17A and FOXP3 was less pronounced in HO, while that of IL-17C was increased demonstrating a more effective Th17 response. IL-17A is one of the main drivers of parasite-mediated pathology, particularly in the formation of granulomatous tissue, a process regulated by IL-10, TGF-β and Treg cell activity. In a previous study, higher TGF-β expression was observed for the HO fish as compared with the TL fish after *M*. *cerebralis* exposure [7]. Reducing IL-17A in this context enhances protective mechanisms by reducing pathology/parasite prevalence [37]. Hence, the induction of IL-17A and FOXP3 in TL suggests reduction of protective mechanisms enhancing pathology/parasite prevalence. Conversely, HO demonstrates more controlled Th17 responses, which helps these fish to resist *M*. *cerebralis*.

The expression of IL-10 was significantly increased in TL at several time points. Studies of *Trichuris muris* and *Trypanosoma cruzi* revealed a key role for IL-10 in limiting a lethal T cell response [41]. Interleukin 17A and IL-10 were among the most strongly upregulated genes in intestines of gilthead sea bream parasitized with *Enteromyxum leei* [42]. IL-10 was also strongly upregulated during clinical PKD in trout [38]. This is in line with recent findings in *Trypanosoma carassii* infected common carp showing Th17 type responses and increasing IL-17A expression [43]. In accordance with this concept, in the current study, the expression of IL-10 increased in TL fish in addition to IL-17A, likely attempt to balance the inflammatory response post exposure to *M*. *cerebralis*. However, HO fish seem to have a more balanced and controlled immune response, that may reflect the protection seen.

The Th17 regulatory cytokines IL-6 and IL-23p19a were analysed to investigate whether they were involved in controlling and modulating IL-17A IL-17C influencing the outcome of *M*. *cerebralis* infection in salmonids as previously suggested [13]. In the current study, the expression of IL-6 increased in TL at several time points. IL-6 is a pleiotropic cytokine with pro- and anti-inflammatory functions. IL-6 activates STAT3 and stimulates the STAT3/SOCS3 pathway, an important negative regulatory response in vivo [44–46]. Trout IL-6 can rapidly induce SOCS3 in RTS-11 cells [33]. The IL-6/STAT3 pathway likely stimulates SOCS3 (IL-6-dependent SOCS3) [45]. On the other hand, SOCS3 specifically prevents activation of STAT3 and selectively blocks signaling by IL-6 [46, 47]. IL-6 has a key anti-inflammatory function in both local and systemic acute inflammatory responses [48]. While, IL-6 has a protective role in many infections, it can be the key to the maintenance of chronic inflammatory conditions [49]. However, a failure to block IL-6 signaling results in a profound anti-inflammatory signal that limits the generation of protective immunity to *T*. *gondii* [50]. Trout infected with *T*. *bryosalmonae* showed upregulation of IL-6, IL-10 and Th17 cytokines in head kidney [34]. Similarly, IL-6, IL-10 and Th17 cytokines increased in TL at multiple time points suggesting the dysregulation of protective immune responses due to expansion of Treg cells. Further, an emerging role of IL-6 in modulating several functions of immune cells has been reported, including T cells, dendritic cells (DC), and macrophages [51]. A novel mechanism of DC-dependent CD4^+^ T cell immune dysfunction was attributed to IL-6 overproduction. IL-6-associated arginase activity downregulates MHC-II expression in DC and suppresses CD4^+^ cell-mediated immunity [52–53]. MHC-II is known to be associated with disease susceptibility/resistance in salmonids [15], although many factors are known to influence its level of expression [54–55]. During WD, arginase expression was modulated [9]. Treg17 cells are induced by IL-6 and TGF-β, and produce IL-17 and IL-10 offering a novel regulatory T cell population for modulating immune responses [30,32]. In the present study, the increased expression of IL-6 in TL could promote Treg17 cells resulting in dysregulation of Th cell-mediated immunity. IL- 6 expression in HO was more moderate than in TL, potentially enabling a more controlled host response. If such a scenario is correct then it would support that the IL-6 pleiotropic function is conserved in fish.

The expression of IL-23 was differentially regulated in HO and TL after exposure to *M*. *cerebralis*. IL-17 secretion is primarily induced in activated CD4^+^ T cells by IL-23. Several studies present suggest that Th17 cytokines are reliant on the IL-23/IL-17 axis for pathogen control at mucosal barriers [25]. Th17-cell differentiation is initiated by IL-6 but IL-23 is necessary for expansion and maintenance of Th17 cells. IL-23 activates STAT3, which has a crucial role in both IL-6- and IL-23-mediated Th17-cell stimulation and is negatively regulated by SOCS3 [56]. In HO fish, the expression of IL-23 was markedly upregulated at 2 dpe associated with the lowest parasite number observed. This suggests that IL-23 orchestrates Th17 responses through activating STAT3 maintaining effective Th17/Treg17 balance by endorsing effector Th17 cells and limiting excessive Treg17 cells in these fish.

The expression of STAT1 was increased at several time points in HO fish. STAT1 is typically regulated by STAT3. Phosphorylated STAT3 can homodimerize, as well as heterodimerize with STAT1, prior to nuclear transport [57]. In accordance with previous observations [10], the expression of STAT3 was differentially regulated between susceptible and resistant rainbow trout strains showing increased values in HO fish. STAT3 plays a key role in immune response signaling and transcriptional regulation and has a critical function in Th17 cell differentiation, which seems to be particularly relevant to the host response against *M*. *cerebralis* invasion as Th17 secreted cytokines can reduce pathogen loads at epithelial and mucosal barriers [20–24]. Since STAT3 increased in HO and decreased in TL fish in the present study, it likely has a critical role in this host-pathogen interaction.

The expression of SOCS3 was upregulated in TL fish. As SOCS3 is key negative regulator of IL-23 signaling and STAT3 activation, it can constrain Th17 differentiation favouring the induction of Treg17 [58–59]. Regardless of whether IL-6 or IL-23 is present, SOCS3 appears to be a critical regulator of Th17 responses attenuating Th17 generation [59]. In TL, the highest expression value for SOCS3 was observed at 2 dpe correlating with the highest relative parasite load compared to HO. This could be a consequence of SOCS3 constraining IL-23 signaling and STAT3 activation, thereby attenuating Th17 differentiation and favouring the induction of Treg17 cells. The expression of SOCS1 was markedly elevated at multiple time points in TL fish, with its highest value also at 2 dpe similar to SOCS3 also correlating with the highest relative parasite load. SOCS1 performs essential roles in maintaining stability and function of Treg cells by inducing FOXP3 expression and IL-10 production [59]. In the present study, in addition to the IL-6-dependent SOCS3 upregulation, the expression of SOCS1 as well as that of IL-10 and FOXP3 was increased in TL fish. On the other hand, the expression of STAT1 and IL-23-activated STAT3 was predominantly elevated in HO fish, underscoring the potential involvement of the STAT3/SOCS3 axis in shaping the susceptible/resistant phenotype.

Such results suggest that the *M*. *cerebralis* evasion strategy may involve suppressing Th17 immunity by inducing Th17-specific Treg17 cells as well as FOXP3 Treg cells in rainbow trout. Different leucocyte populations including T cells and Th cells were isolated from skin of rainbow trout after *M*. *cerebralis* exposure [13]. In the present study, the expression of IL-17A, IL-6, IL-10, SOCS1, SOCS3 as well as FOXP3 were markedly increased in the susceptible rainbow trout. Hence, this suggests the transdifferentiation of Th17 cells into Th17-specific Treg17 and the induction of FOXP3^+^ Treg at epithelial and mucosal barriers in TL fish enhancing their susceptibility to infection. In contrast, HO fish appear to overcome these manipulating strategies, potentially by activating STAT3 via IL-23 to maintain effective Th17 responses. Collectively, our findings suggests that the STAT3/SOCS3 signalling axis is likely conserved in fish and may play a pivotal role in the resistance mechanisms directing WD outcome in rainbow trout.

### Conclusions

In the present study, in TL fish the expression of SOCS1 and the IL-6-dependent SOCS3 increased, whereas the expression of STAT1 and STAT3 decreased at the majority of time points post-infection, likely impacting the ability to execute a protective immune response against *M*. *cerebralis*. SOCS1 and SOCS3 potentially constrain the expression of STAT1 and STAT3 in TL causing Th17/Treg17 imbalance, leaving fish unable to reduce parasite burden or control inflammatory reactions. On the other hand, in HO fish, the expression of STAT1 and IL-23-activated STAT3 may increase the likelihood of maintaining an appropriate Th17/Treg17 balance, thereby enabling fish to limit parasite numbers and contributing to WD resistance. However, investigating nerve tissues, cartilage and systemic responses as well as functional studies are crucial to elucidate the precise roles that the STAT3/SOCS3 axis plays in shaping the Th17/Treg balance and determining WD outcome. Above all, the plasticity of Th17 cells and the role of pleiotropic IL-6 in inducing Treg17 and IL-17A producing FOXP3^+^ Treg cells during parasitic infestation and their involvement in provoking inflammation and host tissue damage warrant further investigation in diseased fish, especially when associated with uncontrolled inflammatory conditions. The knowledge gained by investigating the mechanistic differences between resistant and susceptible rainbow trout strains and by studying the evasion strategies used by *M*. *cerebralis* may pave the way to develop effective management plans to limit the distribution and severity of WD. This will require a combination of complementary approaches such as linkage mapping and gene expression studies, to reveal the genetic variation and mechanisms that underlie WD resistance in rainbow trout.
